# Genome expansion by a CRISPR trimmer-integrase

**DOI:** 10.1038/s41586-023-06178-2

**Published:** 2023-06-14

**Authors:** Joy Y. Wang, Owen T. Tuck, Petr Skopintsev, Katarzyna M. Soczek, Gary Li, Basem Al-Shayeb, Julia Zhou, Jennifer A. Doudna

**Affiliations:** 1 Department of Chemistry, University of California, Berkeley, CA USA; 2 Innovative Genomics Institute, University of California, Berkeley, CA USA; 3 Department of Molecular and Cell Biology, University of California, Berkeley, CA USA; 4 California Institute for Quantitative Biosciences (QB3), University of California, Berkeley, CA USA; 5 Department of Bioengineering, University of California, Berkeley, CA USA; 6 Howard Hughes Medical Institute, University of California, Berkeley, CA USA; 7 MBIB Division, Lawrence Berkeley National Laboratory, Berkeley, CA USA; 8 Gladstone Institutes, University of California, San Francisco, CA USA

**Keywords:** DNA repair enzymes, Bacterial genomics

## Abstract

CRISPR–Cas adaptive immune systems capture DNA fragments from invading mobile genetic elements and integrate them into the host genome to provide a template for RNA-guided immunity^1^. CRISPR systems maintain genome integrity and avoid autoimmunity by distinguishing between self and non-self, a process for which the CRISPR/Cas1–Cas2 integrase is necessary but not sufficient^2–5^. In some microorganisms, the Cas4 endonuclease assists CRISPR adaptation^6,7^, but many CRISPR–Cas systems lack Cas4^8^. Here we show here that an elegant alternative pathway in a type I-E system uses an internal DnaQ-like exonuclease (DEDDh) to select and process DNA for integration using the protospacer adjacent motif (PAM). The natural Cas1–Cas2/exonuclease fusion (trimmer-integrase) catalyses coordinated DNA capture, trimming and integration. Five cryo-electron microscopy structures of the CRISPR trimmer-integrase, visualized both before and during DNA integration, show how asymmetric processing generates size-defined, PAM-containing substrates. Before genome integration, the PAM sequence is released by Cas1 and cleaved by the exonuclease, marking inserted DNA as self and preventing aberrant CRISPR targeting of the host. Together, these data support a model in which CRISPR systems lacking Cas4 use fused or recruited^9,10^ exonucleases for faithful acquisition of new CRISPR immune sequences.

## Abstract (web summary)

CRISPR systems lacking Cas4 can use fused or recruited exonucleases for faithful acquisition of new CRISPR immune sequences.

## Main

Prokaryotes use CRISPR–Cas adaptive immune systems to create a sequential genetic record of infection^11^. Transcription and processing of CRISPR sequence arrays, which consist of short repeats and around 30 bp foreign DNA-derived spacers^1,2,12,13^, yields mature CRISPR RNAs (crRNAs) that guide interference of matching genetic material, protecting the host against recorded sequences^14–19^.

The Cas1–Cas2 integrase drives CRISPR array evolution by selecting and inserting new spacers^3,20,21^. Cas1_4_–Cas2_2_ is a heterohexameric complex that specifically recognizes DNA fragments (protospacers) containing an approximately 30 bp segment with short single-stranded 3′ overhangs^21–23^. In DNA-targeting CRISPR systems, protospacer selection requires a flanking 2–5 bp sequence known as the PAM, which is a key component used to distinguish self from non-self and evade autoimmunity. The PAM is selected during DNA capture, but is removed before host genome integration. Coordinated selection and removal of the PAM ensures Cas interference modules target true invasive elements instead of the host CRISPR array^5,24,25^.

Diverse mechanisms of PAM selection and removal underscore the importance of the PAM for maintaining both adaptive immunity and genome integrity during CRISPR sequence acquisition^8–10,26–28^. In CRISPR systems including type II-B, some type V, and type I-A, I-B, I-C, I-D and I-G, the Cas4 endonuclease performs PAM selection and processing^6,7,26,29,30^. However, around 40% of CRISPR subtypes lack Cas4^8^. In systems lacking Cas4 such as the type I-E system in the common laboratory *Escherichia coli* K12 strain, Cas1 contains a PAM-binding pocket that is believed to participate in protospacer precursor (prespacer) selection^9,23^. However, whether Cas1 cleaves the PAM in a similar manner to Cas4 or relies on host nucleases to perform this function remains unclear^9,23^. Recent in vitro studies identified host exonucleases that have the ability to aid Cas1–Cas2 in prespacer substrate trimming^9,10,31^. Standalone exonucleases such as the DnaQ-like exonuclease class DEDDh are widespread ancillary components that are present in every CRISPR–Cas type^8^. There are also type I-E systems containing a natural Cas2/DEDDh exonuclease fusion^8,28,32^, further implying a functional link between exonucleases and the CRISPR integrase. These systems provide a model for studying coordination between host exonucleases and CRISPR integrases.

Here we reconstitute CRISPR sequence capture, processing and integration by a naturally occurring *Megasphaera* NM10-related Cas2 and DEDDh fusion protein (Cas2/DEDDh) in complex with Cas1 (Cas1–Cas2/DEDDh). We show that Cas1–Cas2/DEDDh preserves the PAM during prespacer processing and the first step of integration. The PAM is removed before completing full integration. The DEDDh active site, rather than Cas1^23^, is responsible for both initial 3′ overhang trimming and PAM removal. This mechanism is distinct from that of Cas4, which cleaves the PAM endonucleolytically, suggesting a divergent role for host exonucleases in PAM processing^9^. The integrase regulates DEDDh exonuclease activity by a ruler-guided, gatekeeping mechanism that coordinates processing and defines the length of integrated DNA. Cryo-electron microscopy (cryo-EM) structures of Cas1–Cas2/DEDDh bound to prespacer DNA with or without the PAM show how Cas1–Cas2 recognizes the sequence and protects it from DEDDh-mediated trimming. Conformational analysis of half-integration structures suggests that, once anchored into the CRISPR array, DNA bending engages the C-terminal region of Cas1, which in turn exposes the PAM for removal, enabling full integration. Our findings provide a general mechanism for exonuclease-assisted PAM processing and demonstrate that CRISPR systems evolved diverse mechanisms to ensure robust immunity against parasitic elements and avoid autoimmunity.

## Cas1–Cas2/DEDDh substrate generation

**Fig. 1:**
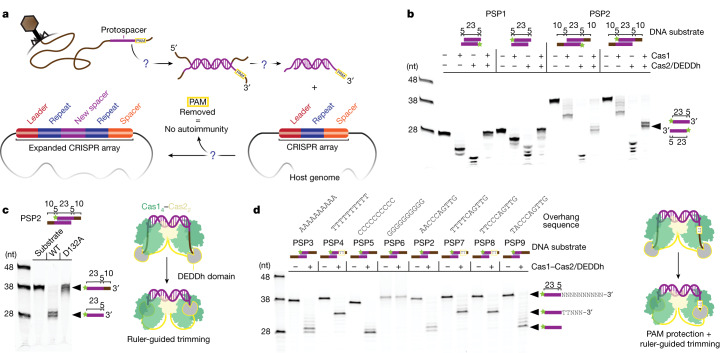
Cas1–Cas2/DEDDh processes prespacers to the correct size for integration and protects a TT PAM. **a**, Open questions in CRISPR adaptation. **b**, Processing of fluorescently labelled prespacer substrates with a 23 bp duplex and different overhang lengths by Cas1–Cas2/DEDDh down to 5–6-nucleotide (nt) single-stranded 3′ overhangs. Prespacer 1 (PSP1), 23 bp duplex with 5-nucleotide 3′ overhangs; prespacer 2, 23 bp duplex with 15-nucleotide 3′ overhangs. The star indicates the 6-carboxyfluorescein label. **c**, WT and mutant Cas1–Cas2/DEDDh (DEDDh(D132A)) prespacer processing. **d**, Processing of substrates with variable 3′ overhangs and the model of observed PAM protection and ruler-guided trimming by the DEDDh domain. Gel source data are provided in Supplementary Fig. 1.

**Extended Data Fig. 1:**
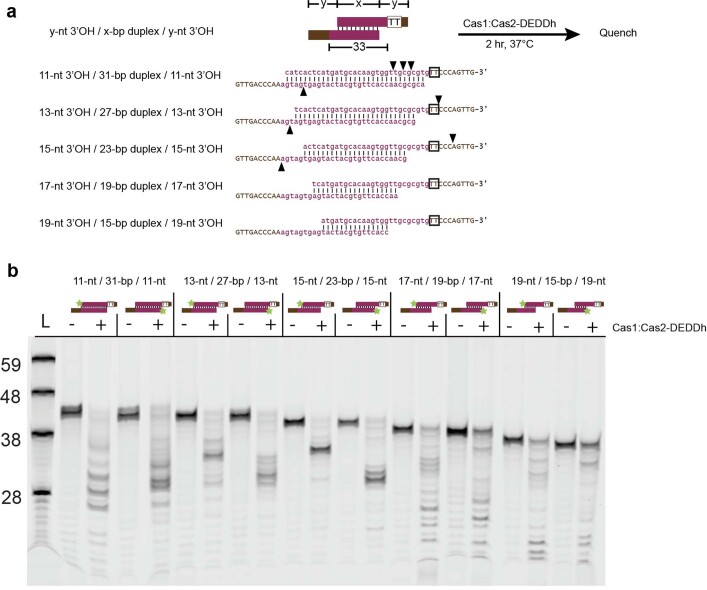
Cas1—Cas2-DEDDh exhibits strict requirement for pre-duplexed 23 bp DNA duplex prespacer for functional ruler-guided trimming. (**a**) Prespacer substrates with varying duplex lengths used in processing assay. The duplex and overhang lengths are indicated, and the TT PAM motif is boxed. The arrowheads indicate specific processing positions as observed from the processing products visualized on the denaturing gel in **b**. (**b**) Processing assay with pre-duplexed prespacer substrates shown in **a**. Substrates are schematized. For gel source data, see Supplementary Fig. 1. This experiment was repeated thrice with similar results.

CRISPR adaptation relies on the recognition, capture and processing of suitable DNA integration substrates from foreign sources (Fig. 1a). These integration substrates (prespacers) require nucleolytic processing to generate fragments of uniform length. To investigate the predicted exonuclease domain of Cas2/DEDDh, we expressed and purified Cas1 and Cas2/DEDDh from a type I-E *Megasphaera* NM10-related CRISPR system and tested DNA substrate processing in vitro (Fig. 1b). The size of spacers in the *Megasphaera* CRISPR array and preferences of the related I-E *E. coli* Cas1–Cas2 integrase suggest the preferred substrate is a 23 bp DNA duplex with 5 nucleotide single-stranded 3′ overhangs^22,23^. To test DNA processing, we assayed Cas1 and Cas2/DEDDh trimming activity using 5′ fluorophore-labelled prespacer substrates containing a 23 bp duplex region and extended single-stranded 3′ overhangs of varying lengths. Cas1 and Cas2/DEDDh each exhibit nuclease activity in isolation, yielding distinct products without apparent functional relevance (corresponding to partial cleavage or complete trimming of the 3′ end, respectively). Only the reconstituted Cas1–Cas2/DEDDh complex generates substrates equivalent in size to spacers in the host CRISPR array (Fig. 1b). Varying the substrate sizes showed that the integrase requires a 23 bp duplex for functional processing (Extended Data Fig. 1a,b). We next tested whether the DEDDh active site is responsible for processing activity by using a catalytically inactive DEDDh mutant (D132A). DNA cleavage assays indicated that the mutant complex does not process prespacers (Fig. 1c). Taken together, these data demonstrate that the complete Cas1–Cas2/DEDDh complex is necessary for prespacer processing and that the DEDDh active site provides the requisite nucleolytic activity.

**Extended Data Fig. 2:**
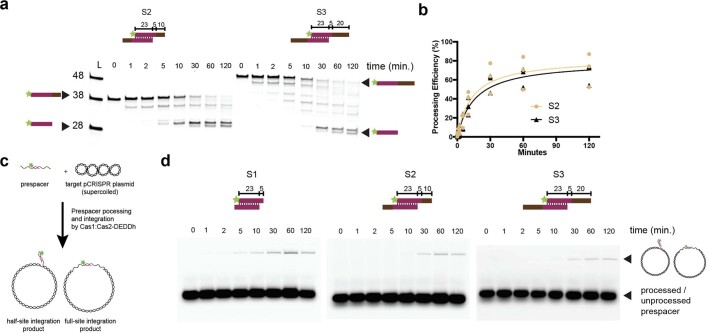
Kinetics of exonuclease trimming and ligation by Cas1:Cas2-DEDDh *in vitro*. (**a**) Time-course reactions of ruler-guided trimming by Cas1:Cas2-DEDDh using substrates S2 and S3 over two hours. (**b**) Quantification of time-course reactions of ruler-guided trimming shown in **a**. Processing efficiency is calculated as the percentage of fully processed product formation at 28-29 nt (n = 3 biologically independent experiments). (**c**) Schematic of *in vitro* ligation assay with integration target pCRISPR plasmid and prespacer substrate. Star indicates 6-carboxyfluorescein (6-FAM) label. (**d**) Time-course reactions of ligation assay with substrates from (b) over 2 h. Note formation of a 6-FAM-labelled pCRISPR plasmid ligation product band. For gel source data, see Supplementary Fig. 1. This experiment was repeated twice with similar results. Source data

Time-course assays suggest similar processing efficiencies for prespacer substrates with varying overhang lengths (Extended Data Fig. 2a–d). Fluorescently labelled prespacers were incorporated into an integration target plasmid (pCRISPR) containing a shortened version of the natural *Megasphaera* CRISPR array. Kinetic analysis implies higher relative integration efficiency with the canonical substrate (23 bp duplex with 5-nucleotide single-stranded 3′ overhangs) compared with prespacers with extended overhangs. Reaction with the canonical substrate generated ligation products after 2 min, while prespacers with extended overhangs required 10 min for detection. Thus, Cas1–Cas2/DEDDh provides a molecular ruler against which DEDDh trims prespacers.

To determine the effect of the PAM, we varied the overhanging region, generating a small prespacer library against which the PAM could be inferred (Fig. 1d). We determined that Cas1–Cas2/DEDDh recognizes 5′-TT in the PAM position. In the absence of a TT PAM, DEDDh trims prespacer strands to the integration-competent size (28 nucleotides). However, the presence of a TT PAM in the correct position (nucleotide positions 29 and 30 relative to the 5′ end) results in partial trimming of the PAM-containing strand, precisely 3 nucleotides away from the PAM, yielding a 33-nucleotide product. We hypothesized that partial trimming was the result of sequestration by a PAM-binding pocket in Cas1^23^.

## PAM binding and prespacer processing

**Fig. 2:**
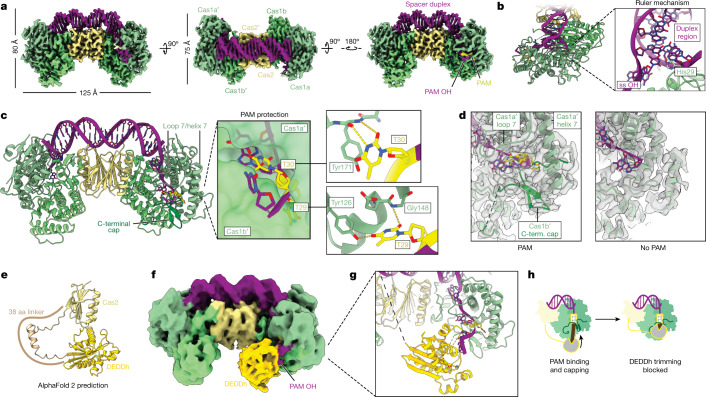
Molecular detail of Cas1–Cas2/DEDDh during prespacer processing. **a**, Orthogonal views of the final cryo-EM densities for Cas1–Cas2/DEDDh bound to a prespacer containing a phosphorothioated TT PAM (threshold, 0.200). **b**, The structure of PAM-bound Cas1–Cas2/DEDDh, depicting one of two His29 residues dictating duplex length. ss OH, single-stranded overhang. **c**, The structure viewed from the PAM side (left). Middle, surface depiction of the cleft between Cas1a′ and Cas1b′. Right, sequence-specific contacts made with each PAM thymine. **d**, Comparison of PAM and non-PAM densities with atomic models overlaid at a threshold of 0.200. Term., terminal. **e**, AlphaFold 2 prediction of the structure of Cas2/DEDDh. aa, amino acid. **f**, Side view of unsharpened cryo-EM density in which DEDDh was resolved (threshold, 0.033). **g**, Hybrid structure containing the DEDDh domain with detail at the PAM–DEDDh interface, with catalytic DEDDh residues shown. The black dashed line represents the unstructured linker between Cas2 and DEDDh domains. **h**, Model for PAM protection.

**Extended Data Fig. 3:**
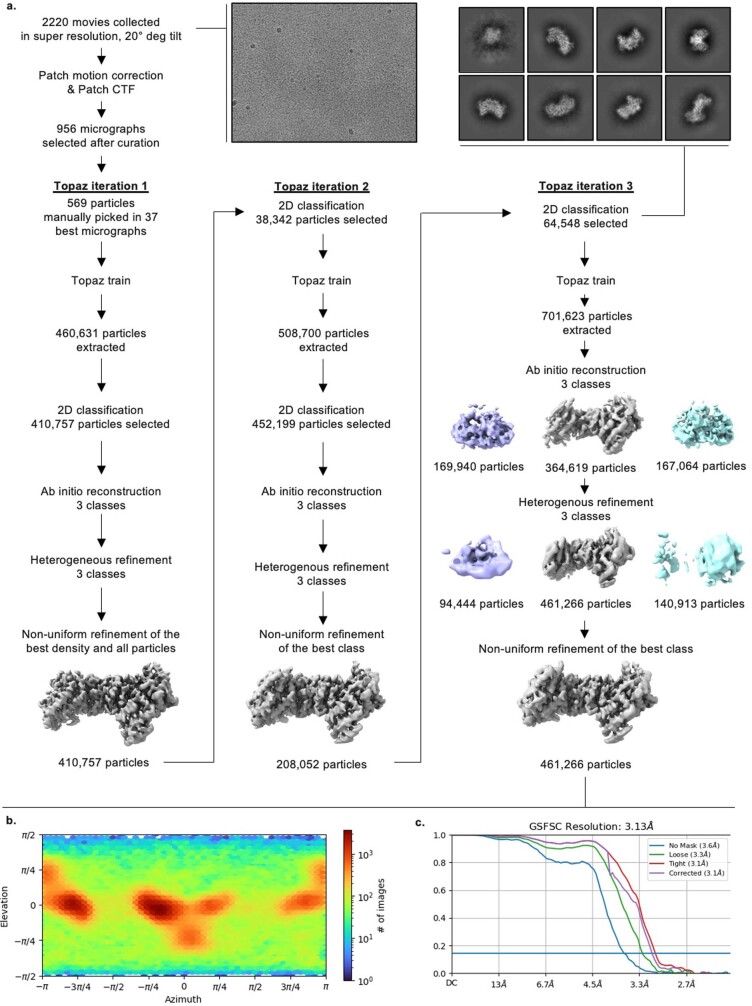
Flow-chart of the cryo-EM single particle reconstruction of the PAM-deficient prespacer bound Cas1-Cas2/DEDDh. (**a**) Map generation pipeline in cryoSPARC consisting of three Topaz training^46^ iterations, including representative 2D class averages and 3D maps resulting from *ab initio* reconstruction and further heterogeneous, non-uniform refinement. (**b**) Orientation distribution of the final set of refined particles. (**c**) Gold standard FSC curve of the atomic model refined to the final cryoSPARC sharp map.

**Extended Data Fig. 4:**
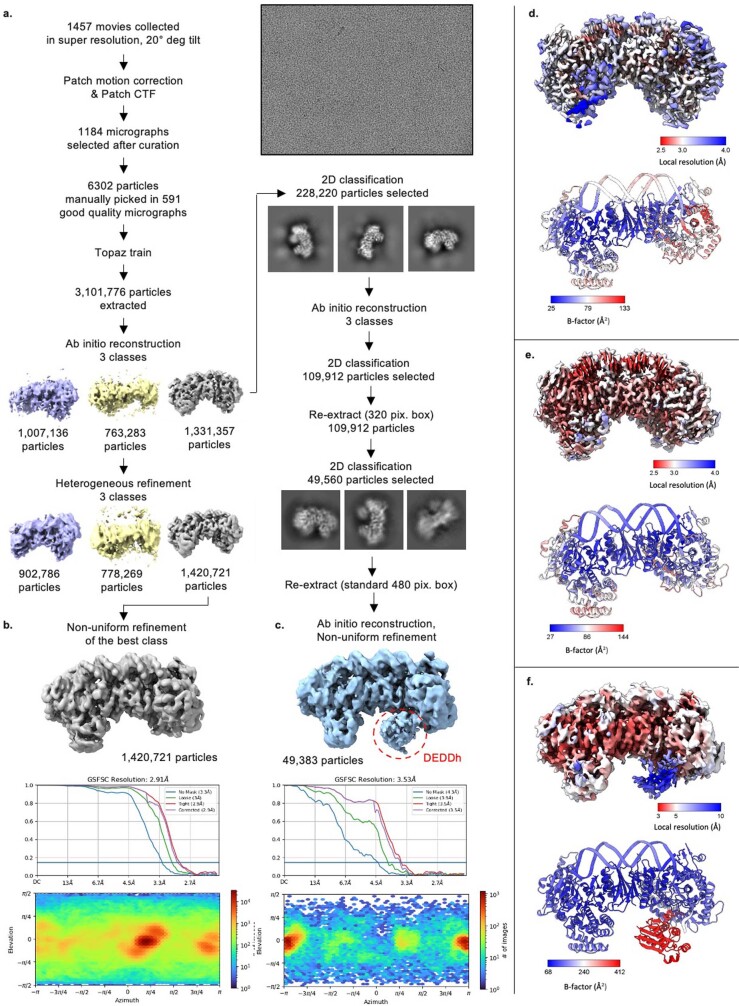
Flow-chart of the cryo-EM single particle reconstruction of the PAM-containing prespacer bound Cas1-Cas2 and resolution of the DEDDh density. (**a**) Cryo-EM data collection parameters and map generation pipeline in cryoSPARC, including representative 2D class averages and 3D maps resulting from *ab initio* reconstruction and further heterogeneous refinement. (**b**) 3D maps, orientation distribution, and gold standard FSC curve of the final cryoSPARC map for the PAM-only (**b**) and DEDDh-containing (**c**) densities. (**d**–**f**) Final sharp maps of Cas1:Cas2-DEDDh bound to prespacers coloured according to local resolution as calculated by cryoSPARC, and the final refined models coloured with B-factors as calculated by Phenix, for: (**d**) PAM-deficient density, (**e**) PAM-containing DEDDh-absent density, and (**f**) PAM-containing, DEDDh-containing density.

We next sought to elucidate the structural basis for prespacer processing and PAM protection. Cryo-EM was used to solve 3.1 Å and 2.9 Å resolution structures of Cas1–Cas2/DEDDh complexed with prespacer substrates with and without a TT PAM, respectively (Fig. 2a–d, Extended Data Table 1 and Extended Data Fig. 3). The *Megasphaera* Cas1–Cas2 retains the canonical heterohexameric architecture^33^, with two Cas1 dimers (denoted Cas1 and Cas1′, a or b subunit) bridged by a central Cas2 dimer (Fig. 2a). A 23 bp duplex sits on the top of the complex, while 5-nucleotide single-stranded 3′ overhangs extend into the clefts formed at opposite Cas1 interfaces. The DEDDh domain could not be resolved in the PAM-absent dataset, presumably due to a high degree of conformational flexibility conferred by the 38 amino acid linker between DEDDh and Cas2, and because all 3′ ends are buried within the complex, protected from exonuclease activity (Fig. 2c). However, in the PAM-containing dataset, the DEDDh domain was resolved by iterative classification and three-dimensional refinement (Methods and Extended Data Fig. 4).

Cas1–Cas2/DEDDh dictates prespacer duplex length with an internal ruler that ensures that spacers are equivalent in length (Fig. 1b,c). Dual Cas1 His29 histidyl residues measure out a 23 bp DNA duplex by π-stacking with the terminal base pairs of the double-stranded region, clasping the prespacer strands and marking the start of the single-stranded 3′ overhang (Fig. 2b). Biochemical processing assays demonstrate that only the 23 bp duplex is both tolerated and trimmed to the integration-competent length (Extended Data Fig. 1a,b).

Processing experiments suggest that the PAM is protected initially from DEDDh-mediated prespacer processing (Fig. 1b–d). To understand how the integrase sequesters the PAM, we performed cryo-EM analysis of the integrase complex bound to a PAM-containing prespacer with a phosphorothioate backbone modification at the predicted site of DEDDh activity, with the intention of stalling processing. Sequence-specific interactions with loop 7 (Asn162 to Asp179) and helix 7 (Met139 to Tyr161) in the Cas1a′ subunit in the resultant density rationalize PAM recognition (Fig. 2c). The first PAM thymine is buried in a pocket in Cas1a′, where hydrogen bonds formed with Tyr126 and Gly148 may enhance binding affinity (Fig. 2c (top right)). The second PAM thymine π-stacks with Tyr171, which positions T30 to hydrogen bond with the Tyr171 backbone amide nitrogen (Fig. 2c (bottom right)). When the PAM is absent, the substrates are fully trimmed, underscoring the necessity of sequence-specific interactions for asymmetric trimming and PAM protection (Fig. 1d). Moreover, a β-hairpin in the C-terminal region of the Cas1b′ ‘caps’ sequestered nucleotides. Loop 7, helix 7 and the C-terminal cap are absent in the PAM-deficient density, suggesting that these structural motifs participate in PAM protection (Fig. 2d).

The DEDDh domain was not visible in the initial PAM-containing structure, raising the question of how the integrase performs ruler-guided trimming of sequestered nucleotides. To resolve the DEDDh domain, we iteratively classified and refined PAM-containing particles and found a density corresponding to DEDDh in a small subset of the total ensemble (Fig. 2f and Extended Data Fig. 4). Key features include a large protrusion only visible on the PAM side of the complex and an extended density attributable to additional phosphorothioate-containing nucleotides 3′ to the PAM (Fig. 2f). As the protuberance was low resolution, the model predicted by AlphaFold 2 for the DEDDh domain was docked into the density (Fig. 2f,g). The resulting hybrid model illustrates dynamics of DEDDh trimming and PAM protection. Catalytic DEDDh residues are poised to exonucleolytically cleave the overhang, but the PAM-binding pocket and the C-terminal loop occlude DEDDh procession, blocking cleavage of the PAM and 2–3 additional nucleotides (Fig. 2h). Despite the high local concentration of non-specific exonuclease relative to the substrate, this process is precise, in concordance with biochemical evidence (Figs. 1b and 2g). A natural consequence of protection is that the PAM must be cleaved downstream.

## PAM trimming after half-integration

**Fig. 3:**
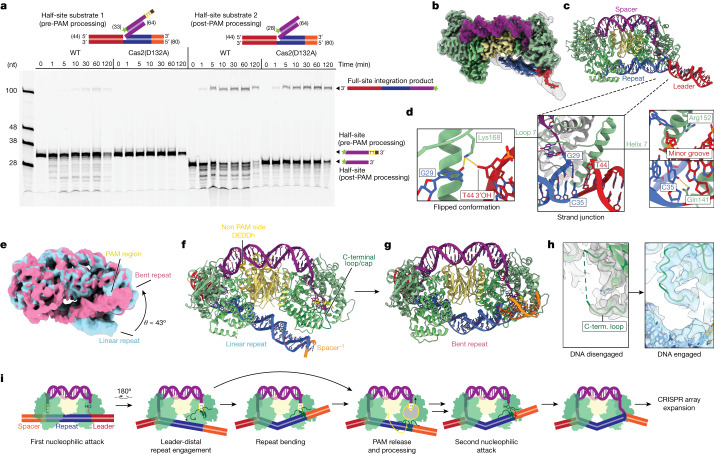
Biochemical and structural analysis of Cas1–Cas2/DEDDh PAM processing. **a**, Integration reactions by WT and mutant Cas1–Cas2/DEDDh (DEDDh(D132A)) half-site integration intermediates. Gel source data are provided in Supplementary Fig. 1. **b**, Unsharpened (grey transparent; threshold, 0.05) and sharp (colour; threshold, 0.19) cryo-EM densities for Cas1–Cas2/DEDDh bound to the PAM-phosphorothioated half-site integration analogue. **c**, The structure of the initial half-site complex. **d**, Details of the junction of leader, repeat and spacer (middle). Left, a second, flipped conformation of G29. Right, helix 7 interactions. **e**, Unsharpened maps of two repeat DNA conformations, linear (blue) and bent (red), both with a threshold of 0.05. **f**, Structure with linear, extended repeat DNA. **g**, Structure of bent repeat DNA. **h**, Comparison of C-terminal loop ordering in the prespacer (left) and linear repeat (right) structures. **i**, Model for PAM gatekeeping facilitated by the C-terminal loop/cap.

Although there is evidence for PAM protection during prespacer processing, the PAM must be removed before insertion into the CRISPR array to avoid autoimmunity. We analysed Cas1–Cas2/DEDDh processing of DNA substrates designed to mimic intermediates of integration into the CRISPR array to determine the mechanism of PAM removal and resolve dynamics of the complex at the integration target site^7,9^. Two substrates that mimic probable half-integration intermediates—the pre-PAM processing intermediate and the post PAM-processing intermediate (Fig. 3a; half-site substrates 1 and 2, respectively)—were synthesized and assayed in reactions with wild-type (WT) and catalytically inactivated DEDDh complexes. Reaction with half-site substrate 1, which contains the unprocessed PAM, resulted in a 100-nucleotide band corresponding to full-site integration (Fig. 3a). The same band was absent when DEDDh was catalytically inactive. Reactions with PAM-deficient half-site substrate 2 yielded the 100-nucleotide full-site integration product for both the WT and dead DEDDh complexes. These data suggest that, in the WT reaction, the PAM is fully removed before full-site integration. The lower intensity of the full-site integration product generated from half-site substrate 1 compared to that of half-site substrate 2 may be a result of inefficient PAM removal, also observed in kinetics assays (Extended Data Fig. 2a,b). The absence of the integrated product strand in the catalytically inactivated DEDDh condition suggests the DEDDh active site executes PAM processing. Notably, PAM processing is necessary for full-site integration, and the integrase generates a precisely defined insertion product size. Thus, non-specific exonuclease activity generates a ladder of ssDNA overhang fragments in the PAM-containing substrate strand, but only one of these fragment sizes is compatible with full-site integration. This single-nucleotide precision is a result of the Cas1–Cas2 ruler, which simultaneously defines the spacer size and acts as a gate that prevents PAM insertion into the CRISPR array (Fig. 3a). As Cas1-mediated PAM protection was observed during prespacer processing, it is reasonable to assume that Cas1 releases the PAM for DEDDh trimming while engaged on the CRISPR array.

**Extended Data Fig. 5:**
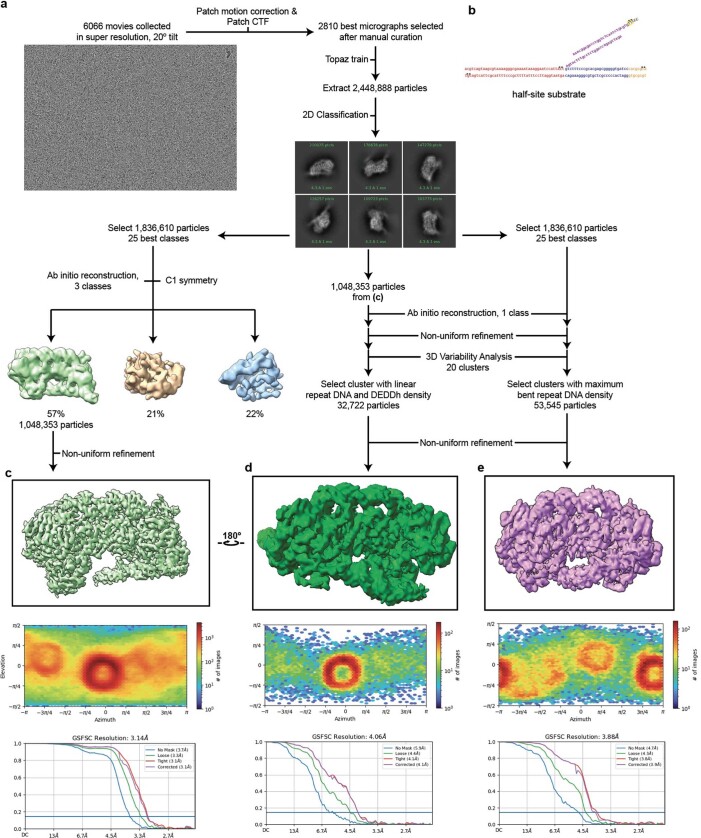
Flow-chart of the cryo-EM single particle reconstruction of the PAM-containing half-site substrate bound to Cas1-Cas2/DEDDh. (**a**) Cryo-EM data collection parameters and map generation pipeline in cryoSPARC, including representative 2D class averages and 3D maps resulting from *ab initio* reconstruction and further heterogeneous refinement. Different particle stacks were used for generation of the high resolution structure (**c**), and 3D variability analysis resulting in linear (**d**) and bent (**e**) density maps. (**b**) Representation of the 4 oligonucleotide half integration construct used in this study. Asterisk indicates a phosphorothioate bond. (**c**–**e**) 3D maps, orientation distribution, and gold standard FSC curve of the atomic model refined to the final cryoSPARC sharp map for each half-site structure.

**Extended Data Fig. 6:**
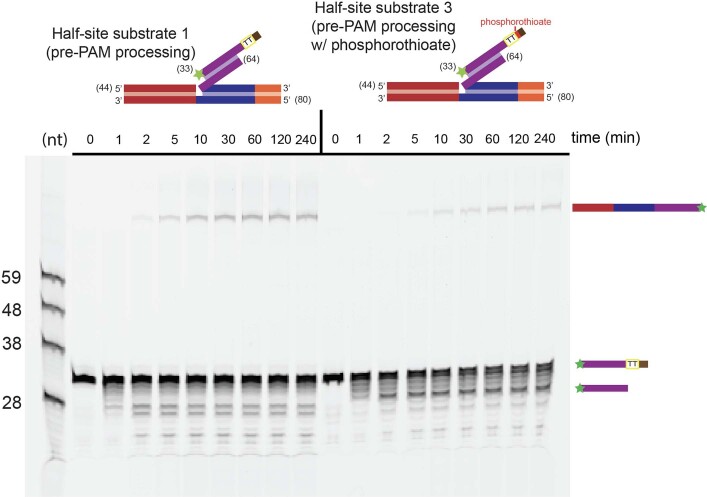
Incomplete resistance of phosphorothioate PAM nucleotides to DEDDh. Processing assay (4 h) with substrates shown in above. Substrates are schematized and TT PAM is indicated, along with phosphorothioation. Processing products are depicted on the right. For gel source data, see Supplementary Fig. 1. This experiment was repeated twice with similar results.

Evidence for DEDDh involvement in both prespacer processing and PAM cleavage led us to examine which molecular cues prompt Cas1 to relinquish the PAM for digestion. Aiming to visualize DEDDh trimming and conformational changes in Cas1, we used cryo-EM to characterize Cas1–Cas2/DEDDh in complex with a DNA half-site analogue containing phosphorothioate linkages at the PAM positions (Fig. 3b and Extended Data Fig. 5a–c). Neither the initial 3.1 Å density nor any heterogeneous states detected during cryo-EM data processing had density corresponding to the DEDDh domain on the PAM side of the complex (Fig. 3b). However, DEDDh was observed on the non-PAM side during DNA conformational analysis. We speculate that, in agreement with biochemical data, only the DEDDh domain can trim the PAM, and PAM trimming activity is required for full integration (Fig. 3a). Furthermore, the DEDDh PAM-trimming state may be transient. Phosphorothioate modifications only partially protected the PAM, which may disfavour resolution of the active DEDDh domain at the half-site (Extended Data Fig. 6).

The 3.1 Å half-site structure reveals interactions at the first integration strand junction (Fig. 3b–d and Extended Data Table 1). After the initial nucleophilic attack, Cas1–Cas2 induces bending of the leader-repeat target DNA^34^. This deformation originates at the nick site, positioning repeat DNA between the leader-distal and -proximal Cas1 active sites. Inspection of the strand junction (Fig. 3d (middle)) reveals interactions with the first base pair of the CRISPR repeat dictated by loop 7 and helix 7 of Cas1a′. The spacer-ligated first CRISPR repeat guanine G29 was found in two approximately equivalent conformations. In the first conformation, G29 forms a canonical base pair with C35 (Fig. 3d (middle)). In the second conformation, G29 flips upwards, making a specific contact with loop 7 lysine Lys168. The lysine also contacts the 3′ hydroxyl of the leader (Fig. 3d (left)). Gln141, which sits at the base of helix 7, also makes a nucleobase-specific contact with C35, the first bottom repeat nucleotide. Helix 7 is well positioned to insert into the minor groove of the leader DNA, but no nucleobase-specific contacts were obvious (Fig. 3d (right)). Specific contacts with CRISPR array nucleotides probably have a functional role in targeting, as previously observed^20,21,35^.

The 3.1 Å half-site complex reconstruction contains density corresponding only to the leader-proximal region of the CRISPR repeat (Fig. 3b,c). To probe the dynamics at the leader-distal region, where PAM processing and subsequent full integration occur, we performed three-dimensional variability analysis (3DVA) of the particle set (Extended Data Fig. 5a,d,e)^36^. 3DVA revealed heterogeneity in the location of the repeat/spacer end, with the CRISPR repeat DNA oscillating between linear and bent conformations (Fig. 3e and Supplementary Video 1). The DEDDh domain was visible only in the linear conformation (Supplementary Video 2). Isolation and refinement of particle clusters representing maxima of the reaction coordinate gave linear and bent reconstructions at resolutions of 4.1 Å and 3.9 Å, respectively (Fig. 3e–g).

**Extended Data Fig. 7:**
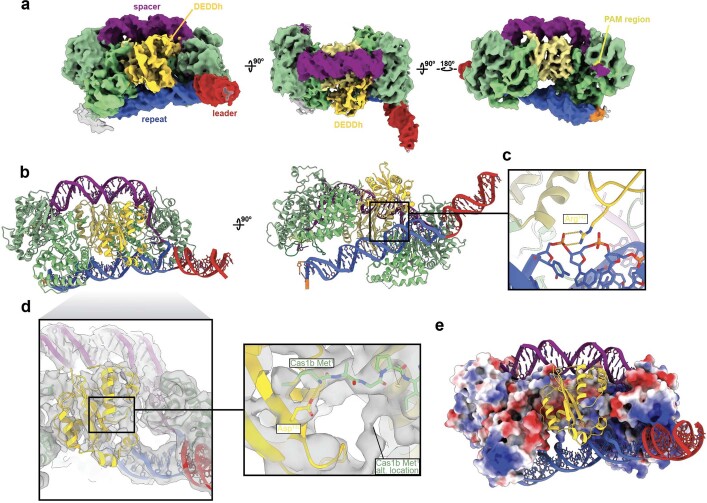
Molecular detail of Cas1:Cas2-DEDDh in the linear CRISPR repeat DNA conformation. (**a**) Orthogonal views of the final sharpened cryo-EM densities for Cas1:Cas2-DEDDh bound to half-site DNA intermediates containing a phosphorothioated TT PAM, coloured to demonstrate domain locations. (**b**) Non-PAM side and bottom views of Cas1:Cas2-DEDDh bound to half-site intermediate DNA. (**c**) Detail depicting potential interaction between Arg^142^ of DEDDh and CRISPR repeat DNA phosphate backbone. (**d**) Non-PAM side view of the DEDDh domain with the sharp map superimposed (threshold: 0.05). Right, detail in the catalytic pocket of DEDDh, with Asp^132^ shown in close proximity to an extension attributed to the N-terminus of Cas1b. (**e**) Cas1:Cas2-DEDDh linear structure, with protein surfaces except the DEDDh domain coloured according to electrostatic potential.

In the linear structure, a Cas1b′ C-terminal loop (Leu279 to Ser293) rich in charged residues is positioned near to the major groove adjacent to the second integration target site (Fig. 3f). The corresponding density is absent in the 3.1 Å half-site and 2.9 Å prespacer-bound structures, indicating that this loop participates in engagement with the CRISPR repeat on the PAM side (Figs. 2c and 3g,h). A C-terminal cap, which follows the C-terminal loop, protects the PAM and adjacent nucleotides from trimming by DEDDh (Fig. 2h). Notably, the DEDDh domain was visible in the linear structure, but on the non-PAM side of the complex, where no overhang trimming occurs (Fig. 3f). The exonuclease sits in a cavity formed by the interface of the CRISPR repeat DNA, Cas1a/b′ and Cas2, where it appears to contact the repeat DNA backbone and the N terminus of Cas1b (Fig. 2f and Extended Data Fig. 7). These interactions may beneficially constrain bending of the second integration target site or prevent *trans* activity.

The bent structure features a pronounced kink in the centre of the repeat region (Fig. 3g). Disruption of a single A–T base pair and DNA unwinding appear to accommodate the strain induced by this pitch, although this assignment was made with low confidence owing to the low local resolution at the bending site. Bending in the centre of the CRISPR repeat symmetrizes the integration complex and draws the repeat/spacer junction towards the Cas1a′ active site. Although PAM nucleotides are still present, probably due to their cleavage-blocking phosphorothioate modifications, the overhang nucleotides typically sequestered during ruler-guided trimming are absent, suggesting that 3′ trimming occurs in an intermediate step between the linear and bent states. Only the first three nucleotides under the C-terminal loop of Cas1b′ could be assigned, and the C-terminal cap density was largely unstructured. These observations, combined with the proximity of the second integration target site in the bent structure, suggest that engagement of the CRISPR DNA by the C-terminal loop induces uncapping of previously sequestered PAM nucleotides, followed by DEDDh-mediated or host-exonuclease-mediated trimming of the exposed 3′ end to the ruler-defined length. Once the PAM is trimmed, full integration occurs (Fig. 3a). Structural and biochemical analyses imply a general mechanism of sequential PAM protection and cleavage, or PAM gatekeeping, which ensures that PAM-deficient protospacers integrated into the CRISPR array are marked as self and are equal in size (Fig. 3i).

## CRISPR array integration reconstitution

**Fig. 4:**
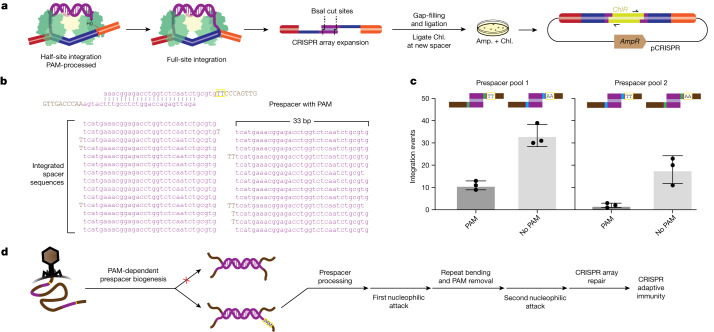
In vitro reconstitution of Cas1–Cas2/DEDDh-mediated full-site integration. **a**, Schematic of in vitro reconstitution of full-site integration. Amp., ampicillin; chl., chloramphenicol. **b**, Fully integrated spacer sequences from a prespacer containing the PAM. Lowercase bases match the prespacer sequence. Uppercase bases are additional, untrimmed nucleotides. **c**, The number of integration events arising from an equimolar pool of prespacers with or without the PAM. Prespacers are distinguished by internal barcodes, which were swapped to remove sequence bias. Data are mean ± s.d. of three independent biological replicates (*n* = 95 colonies). **d**, Proposed general timeline of CRISPR adaptation. Source data

**Extended Data Fig. 8:**
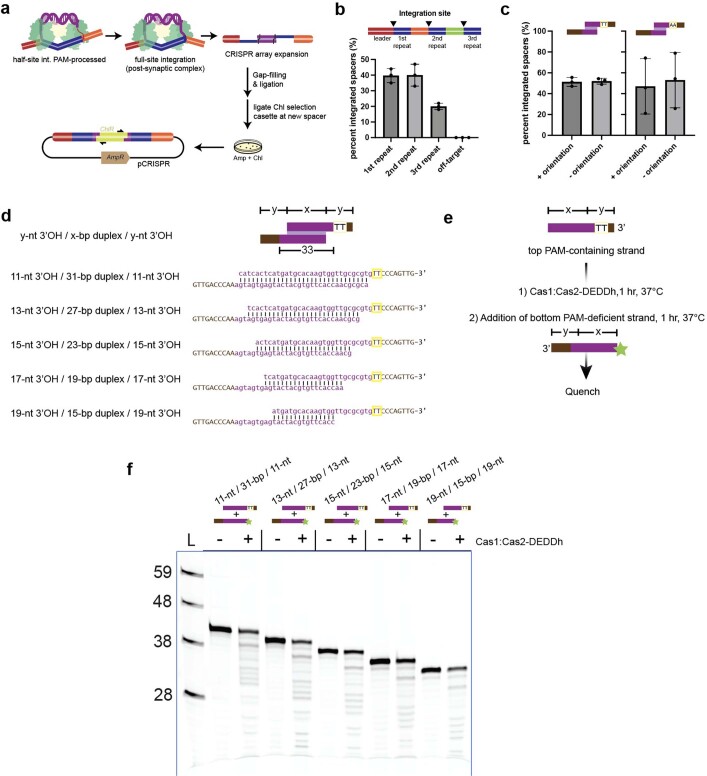
Integration site preference, orientation bias, and duplex requirements of Cas1—Cas2-DEDDh. (**a**) Schematic depicting experimental workflow for full-site integration reconstitution. (**b**) Integration events at each integration site from sequenced clones. Integration sites are depicted by arrows at repeat borders. The mean and standard deviation of three independent biological replicates are shown (n = 95 colonies). (**c**) Left, orientation of spacer insertion from prespacer containing PAM motif, + orientation orients original TT PAM containing end toward the leader, − orientation orients original TT PAM containing end away from the leader. Right, Orientation of spacer insertion from control prespacer without PAM motif (the TT PAM from left is replaced with AA). The mean and standard deviation of three independent biological replicates are shown (n = 95 colonies). (**d**) Prespacer substrates with varying duplex lengths used in processing assay. The duplex and overhang lengths are indicated, and the TT PAM motif is boxed. (**e**) Schematic of processing assay with stepwise addition of the top and bottom prespacer strands of the substrates shown in **d**. The unlabelled top PAM-containing strand is incubated first with Cas1:Cas2-DEDDh followed by delayed addition of the labelled bottom PAM-deficient strand. (**f**) Processing assay with stepwise addition of the top and bottom prespacer strands of the substrates shown in **a**. For gel source data, see Supplementary Fig. 1. This experiment was repeated twice with similar results. Source data

To determine how PAM sequence recognition and gated removal ensures accurate DNA integration, we reconstituted CRISPR substrate integration in vitro. An unprocessed prespacer (23 bp duplex with 15 nucleotide single-stranded overhangs) containing the TT PAM was combined with Cas1–Cas2/DEDDh and pCRISPR. Prespacer substrates encoded BsaI restriction sites in the duplex region to enable insertion of a chloramphenicol-resistance gene. After transformation, only pCRISPR with full prespacer integration confers survival in a double selection assay^37^ (Fig. 4a). Fully integrated sequences provide additional evidence that Cas1–Cas2/DEDDh completes both prespacer processing and integration into the CRISPR array (Fig. 4b). The complex is specific for the CRISPR array and all integration events occur at repeat borders (Extended Data Fig. 8b). However, integration occurs at all three repeats present in the array, without specificity for the leader-proximal repeat, as seen in many CRISPR systems in vivo^1,38^ and consistent with structural data (Fig. 3d). Excess 3′ overhangs are trimmed to within 1–2 nucleotides of the expected length and the PAM is absent in all integrated sequences (Fig. 4b), in agreement with evidence at the level of the half-site (Fig. 3a,i). Reconstitution experiments provide complementary evidence for an alternative mechanism for PAM processing compared with Cas4, which uses a sequence-specific mechanism to cleave the PAM endonucleolytically^7,29^.

**Extended Data Fig. 9:**
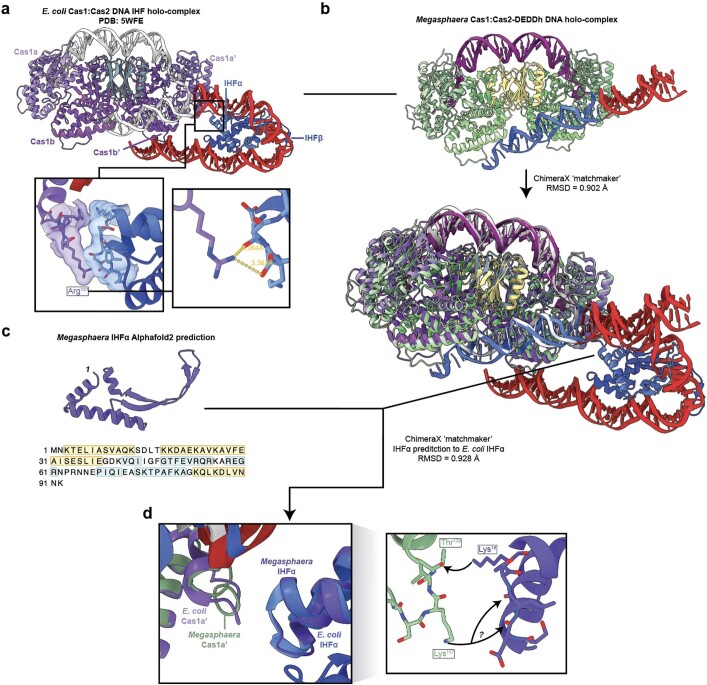
Comparison of Cas1:Cas2-DEDDh bound to the half-site intermediate with the complete *E. coli* IHF-containing integration complex. (**a**) *E. coli* Cas1:Cas2 DNA IHF holo-complex (PDB: 5WFE). Subunit identities are indicated above. Inset boxes highlight the interface between Cas1a and IHF. (**b**) Representation of the pipeline used to compare *Megasphaera* and *E. coli* integrases. The experimental structure solved in this work was superimposed with the *E. coli* complex using ChimeraX matchmaker^54^. (**c**) The sequence of a *Megasphaera* IHFα ortholog was found by protein BLAST of *E. coli* IHFα sequence in the *Megasphaera* sp. An286 genome (Taxon ID: 1965622). The AlphaFold 2 sequence of the highest confidence hit is shown along with the protein sequence, with secondary structures indicated. (**d**) The *Megasphaera* IHFα was superimposed with the *E. coli* IHFα to approximate the interface in a hypothetical *Megasphaera* complex. Inset shows the structures overlaid (left) and with only *Megasphaera* proteins shown (right). Right, potential interactions at the interface of *Megasphaera* Cas1 and IHFα.

Although it was hypothesized that delayed PAM trimming aids the complex in orienting the prespacer for integration^7,9^, no orientation bias was observed in vitro (Extended Data Fig. 8c). Although Cas1–Cas2/DEDDh alone is able to distinguish between the PAM and non-PAM sides of the prespacer (Figs. 1d and 2c), it appears that the complex alone cannot discern the leader- and spacer-side of the repeat, consistent with cryo-EM results of the half-site intermediate, which show no sequence specificity for the leader (Fig. 3d). We suspect that the complex requires additional host factors to correctly orient spacers in vivo. In *E. coli*, integration host factor (IHF) directs the first nucleophilic attack to the leader-side of the repeat through specific contacts with the leader sequence^34,39^. Superimposition of the half-site structure and a *Megasphaera* IHF orthologue onto a structure of the complete IHF-containing integration holo complex further implicates the participation of a directing host factor (Extended Data Fig. 9). In vivo, the system may have higher specificity for the leading integration target site and use delayed PAM processing as the basis for determining the orientation of integration, as is the case in other CRISPR systems^7,9,10^.

To assess the effect of the PAM on integration efficiency, an equimolar mixture of PAM-deficient and PAM-containing prespacers, each containing a pair of identifying internal barcodes, was tested for full-site integration. Notably, out of 95 sequenced colonies, we observed significant enrichment (around threefold) of integration events from the PAM-deficient prespacer (Fig. 4c). To account for biases resulting from the internal barcode sequences, we generated a second prespacer pool, in which barcode pairs were swapped. The second pool also exhibited a significant preference for the PAM-deficient prespacer (Fig. 4c). Lower integration efficiency from the PAM-containing prespacer in vitro may stem from additional steps that are required for PAM removal (Fig. 3i). Moreover, PAM removal is observed after full-site integration, and all spacer sequences are selected according to PAM presence (Fig. 4b). The reduced apparent efficiency of PAM-containing prespacer insertion in vitro therefore suggests PAM recognition in vivo occurs upstream, during the biogenesis of substrates bound for CRISPR adaptation (Fig. 4d).

Integration reconstitution experiments with pooled prespacers suggest that the integrase may select substrates before prespacer processing, ensuring PAM presence (Fig. 4c). We were interested in whether the integrase demonstrates similar stringency for another substrate feature—the canonical prespacer duplex. Accordingly, we tested Cas1–Cas2/DEDDh processing after stepwise addition of the PAM complementary strand. After incubation of Cas1–Cas2/DEDDh with a single-stranded PAM-containing strand, the labelled PAM-deficient strand was added. In all of the reactions, even for the prespacer strands with the tolerated 23-nucleotide complementarity region (Fig. 1b), non-specific processing of the labelled strand occurs (Extended Data Fig. 8d–f). Thus, Cas1–Cas2/DEDDh probably performs ruler-guided trimming when the 23 bp prespacer is preduplexed and not after delayed addition or search for the complementary strand. The strict requirement for substrate size, strandedness and PAM presence has implications for open questions in CRISPR substrate biogenesis.

## Discussion

**Extended Data Fig. 10:**
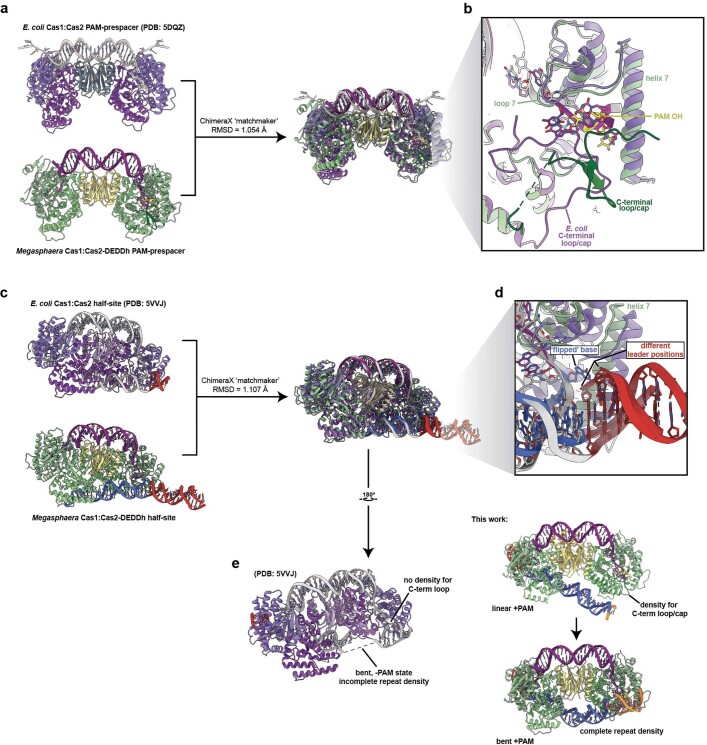
Comparison of Cas1:Cas2-DEDDh to *E. coli* Cas1:Cas2 structures. (**a**) *E. coli* Cas1:Cas2 bound to a PAM-containing prespacer (PDB: 5DQZ) was compared to *Megasphaera* Cas1:Cas2-DEDDh bound to a PAM-containing prespacer. The experimental structure solved in this work was superimposed with the *E. coli* complex using ChimeraX matchmaker^54^. (**b**) Inset showing detail of the superimposition at the PAM binding site. The C-terminal loop in *E. coli* Cas1:Cas2 is oriented differently but covers the PAM in an analogous manner. (**a**) *E. coli* Cas1:Cas2 bound to a PAM-absent half-site DNA (PDB: 5VVJ) was compared to *Megasphaera* Cas1:Cas2-DEDDh bound to a PAM-containing half-site DNA. (**d**) Inset depicting differences at the integration strand junction. (**e**) Comparison of the unintegrated side of both holoenzymes.

Efficient CRISPR adaptive immunity requires coordination between the CRISPR integrase and host nucleases^9,10^. In this study, we describe mechanisms of prespacer processing and integration in a naturally occurring Cas1–Cas2/DEDDh complex. The trimmer-integrase uses an alternative PAM-processing mechanism compared with the well-studied Cas4 endonuclease^7,30^. Previously, it was unclear how systems lacking Cas4 process and integrate substrates. Our data suggest that one evolutionary solution to the problem of selecting, protecting and then removing the PAM is to use Cas1 rather than an accessory protein for initial PAM protection. Sequestration of defined prespacer sizes through substrate gatekeeping ensures that the PAM is present and that its cognate spacer is functional (Fig. 1d). Once the PAM-containing prespacer is anchored to the host CRISPR array, the PAM is released by the Cas1 gate and is promptly removed. We provide a mechanism explaining which structural cues lead to PAM uncapping and removal (Fig. 3i). Binding and bending of leader-distal repeat DNA may lead to disengagement of the C-terminal cap, which covers and protects nucleotides. DEDDh completely digests the released PAM, generating substrates of the correct size and positioned for second nucleophilic attack. This sequence of events ensures that the PAM side integrates second. Bending and unwinding may also aid in the melting of the repeat strand, which is required for resolution of the post-synaptic complex and concomitant repeat duplication^40^. Although the high effective local concentration of exonuclease with respect to the bound prespacer conferred by the Cas2/DEDDh fusion in this system probably improves efficiency, we imagine that host exonucleases can function similarly in *trans*. Cas1–Cas2/DEDDh serves as a general model describing the role of accessory exonucleases, including those that are not fused to the nuclease, in the diverse CRISPR systems lacking Cas4 (comparisons to *E. coli* Cas1–Cas2 are shown in Extended Data Fig. 10).

Recent studies indicated that accessory proteins can coordinate with Cas1–Cas2 to process prespacers. DnaQ and ExoT have previously been shown to process PAM-containing substrates asymmetrically when provided in concert with Cas1–Cas2 in vitro^9,10^, establishing the directionality of integration. Recent in vivo research demonstrated that other accessory exonucleases can substitute for DnaQ and ExoT activities to carry out prespacer processing^31^. These findings suggest that Cas1–Cas2 can flexibly coordinate with various accessory proteins. As a general model, Cas1–Cas2/DEDDh provides insights into the elegant mechanism by which non-specific processing enzymes and Cas1–Cas2 preserve the self versus non-self distinction. These findings advance our understanding of how prespacers are processed and selected for spacer acquisition. We anticipate that our results will be applicable to CRISPR-based technologies that seek to repurpose Cas1–Cas2 for molecular recording and information storage, applications challenged both by reliance on host factors such as exonucleases and by uncertainty in prespacer selection^41–43^.

Although this report represents an advance in our understanding of downstream steps, the upstream biogenesis of CRISPR substrates remains unclear. Unexpectedly, experimental data suggest lower integration efficiency from PAM-containing prespacers and a preference for preformation of the canonical duplex^22^. These results weaken the ‘complement search’ model for prespacer biogenesis, which suggests single-stranded DNA derived from foreign sources are captured independently by the integrase complex. Alternatively, Cas1–Cas2 may recognize PAM-containing prespacer-like motifs as DNA reanneals behind repair complexes implicated in CRISPR adaptation such as RecBCD and AddAB, as was recently suggested^33^. The precise mechanistic details of this proposal are unclear. Future experiments might use the compact trimmer-integrase presented here to investigate open questions in CRISPR substrate biogenesis and achieve total in vitro reconstitution of naive CRISPR adaptation.

## Methods

### Plasmid construction and DNA substrate preparation

To make the target integration plasmid pCRISPR, the leader and the first three repeats and spacers of the CRISPR array were ordered as two DNA fragments, which were amplified by PCR and inserted into the pUC19 backbone by Gibson assembly. DNA oligos used in this study were ordered from Integrated DNA Technologies. Prespacers and the half-site substrates were formed by heating at 95 °C for 5 min and slow cooling to room temperature in HEPES hybridization buffer (20 mM HEPES, pH 7.5, 25 mM KCl and 10 mM MgCl_2_). For the half-site substrate, hybridization was performed with a 1.5-fold excess of the two shortest strands and a 1.25-fold excess of the second-largest strand and purified on an 8% native PAGE gel. Sequences of cloning primers and DNA substrates are shown in Supplementary Table 2.

### Cloning, expression and purification

The *Megasphaera* NM10-related *Cas1* and *Cas2-DEDDh* genes were codon-optimized for *E. coli* expression, ordered as G-blocks, PCR-amplified and cloned separately into a pET-based expression vector with an N-terminal 10×His-MBP-TEV tag. After transformation into chemically competent Rosetta cells, cells were grown to an optical density at 600 nm of around 0.6 and induced overnight at 16 °C with 0.5 mM isopropyl-β-d-thiogalactopyranoside. Cells were collected and resuspended in lysis buffer (20 mM HEPES, pH 7.5, 500 mM NaCl, 10 mM imidazole, 0.1% Triton X-100, 1 mM Tris (2-carboxyethyl)phosphine (TCEP), Complete EDTA-free protease inhibitor (Roche), 0.5 mM phenylmethylsulfonyl fluoride (PMSF) and 10% glycerol). After lysis by sonication and clarification of the lysate by centrifugation, the supernatant was incubated with Ni-NTA resin (Qiagen). The resin was washed with wash buffer (20 mM HEPES, pH 7.5, 500 mM NaCl, 10 mM imidazole, 1 mM TCEP and 5% glycerol) and the protein was eluted with wash buffer supplemented with 300 mM imidazole. After overnight digestion with TEV protease, the salt concentration was diluted to 300 mM NaCl using ion-exchange buffer A (20 mM HEPES, pH 7.5, 1 mM TCEP and 5% glycerol) and run through a tandem MBPTrap column (GE Healthcare) and HiTrap heparin HP column (GE Healthcare) to remove the MBP and bind the protein onto the heparin column. The protein was eluted with a gradient from 300 mM to 1 M KCl, concentrated and purified on the Superdex 200 (16/60) column with storage buffer (20 mM HEPES, pH 7.5, 500 mM KCl, 1 mM TCEP and 5% glycerol). The same purification protocol was used for Cas1 and Cas2/DEDDh (WT and D132A mutant). The sequences of the proteins are provided in Supplementary Table 1.

### Processing assays

Processing assays were conducted in integration buffer (20 mM HEPES, pH 7.5, 125 mM KCl, 10 mM MgCl_2_, 1 mM DTT, 0.01% Nonidet P-40 and 10% DMSO). Cas1 (4 μM) and Cas2/DEDDh (2 μM) were precomplexed for 30 min at 4 °C before addition of fluorescent DNA substrate (312.5 nM) and reacting for 2 h at 37 °C. The reaction was quenched by addition of 2 vol quench buffer (95% formamide, 30 mM EDTA, 0.2% SDS and 400 μg ml^−1^ heparin) and heating at 95 °C for 4 min, before analysis on a 14% urea–PAGE gel. Reactions were visualized using the Typhoon FLA gel imaging scanner and quantification of intensities was performed using ImageQuantTL (v.8.2). The percentage processing activity was quantified as the ratio of the final product band intensity to the total intensity of all bands in the lane.

### Cryo-EM data acquisition

Cas1–Cas2/DEDDh DNA complexes were formed by mixing 50 µM Cas1, 50 µM Cas2/DEDDh, and 12.5 µM prespacer or half-site DNA, and dialysing for 2 h using a Slide-A-Lyzer MINI Dialysis Device at room temperature. The complex was concentrated to varying concentrations of Cas1–Cas2/DEDDh (Extended Data Table 1) and purified over the Superose 6 Increase 10/300 GL column. The samples were frozen using the FEI Vitrobot Mark IV, cooled to 8 °C at 100% humidity. Depending on the sample (Supplementary Table 1), either carbon 2/2 300 mesh C-flat grids (Electron Microscopy Sciences CF-223C-50) or 1.2/1.3 300 mesh UltrAuFoil gold grids (Electron Microscopy Sciences, Q350AR13A) were glow discharged at 15 mA for 25 s using PELCO easyGLOW. In all cases, a total volume of 4 μl sample was applied to the grid and immediately blotted for 5 s with a blot force of 8 units. Micrographs were collected on the Talos Arctica operated at 200 kV and ×36,000 magnification (1.115 Å pixel size), in the super-resolution setting of the K3 Direct Electron Detector. Cryo-EM data were collected using SerialEM (v.3.8.7). Images were obtained in a series of exposures generated by the microscope stage and beam shifts.

### Cryo-EM data processing

All datasets were collected with varied tilt angle, number of videos and defocus range (Supplementary Table 1 and Extended Data Figs. 3–5). Data processing was performed in cryoSPARC (v.3.2.0, v.3.3.1 and v.4.1.1)^44^. Videos were corrected for beam-induced motion using patch motion correction, and contrast transfer function parameters were calculated using patch CTF.

The PAM-deficient prespacer-bound Cas1–Cas2/DEDDh map was obtained through an iterative process. In the first round, 569 particles were picked manually from 37 micrographs and submitted for Topaz training^45^. The resulting Topaz model was used to pick particles from the micrographs, and a total of 460,631 particles was extracted with a bin factor of 2, and applied to 2D classification. After selecting the best classes, 410,757 particles were used for ab initio reconstruction and subsequent heterogenous refinement, with three classes. All of the particles were used for non-uniform map refinement^46^, and an initial complex map was obtained. After 2D classification of particles from the initial non-uniform refinement model, 38,342 particles from the classes with isotropic orientations were selected and processed for the second round of Topaz training. A new Topaz model was used with a total of 956 curated micrographs, and the entire process was repeated twice with particles from the best heterogeneous refinement class for subsequent non-uniform refinement and Topaz training. The final map with the best electron density for the PAM-deficient prespacer bound Cas1–Cas2/DEDDh complex was obtained from 461,266 particles and was refined with non-uniform refinement to 3.1 Å.

For the PAM-containing prespacer-bound Cas1–Cas2/DEDDh, a single round of Topaz training was applied. After the initial exposures curation, which yielded 591 best-quality micrographs, 6,302 particles were manually picked and processed for the Topaz training job. The Topaz model was applied to an expanded set of 1,184 curated micrographs, and resulted in extraction of 3,101,776 particles. After ab initio reconstruction and heterogenous refinement of the particles, with three classes, the 1,420,721-particle set constituting the best class were processed with non-uniform refinement. As a result, a 2.9 Å density for PAM-containing prespacer bound Cas1–Cas2 complex was obtained.

For resolving the DEDDh density in the latter dataset, the ab initio class particles used for the latter density reconstruction, 1,331,357 in total, were applied to a 2D classification job, and 228,220 particles were selected in classes with apparent DEDDh density. After ab initio refinement with three classes, particles from the best class were processed for another round of 2D classification, and 109,912 particles with more pronounced DEDDh density were selected, and re-extraction was performed with a 320 pixel box size (in all other cases, 480 pixel boxes were used for the extraction jobs). As a result of the final 2D classification round, 49,560 particles with the best DEDDh density were selected, re-extracted with standard box dimensions and processed for ab initio refinement, with one class and non-uniform refinement. As a result, a 3.5 Å complex map with the DEDDh exonuclease density was obtained, with a total of 49,383 particles used for reconstruction.

For half-site DNA-bound Cas1–Cas2/DEDDh, the Topaz model from the PAM-containing prespacer was applied to 2,810 micrographs selected after manual curation. The 2,448,888 resultant particles were subdivided using 2D classification, and the 25 best classes were selected, resulting in 1,836,610 particles. These particles were processed for ab initio reconstruction with three classes. The best class containing 1,048,353 particles was refined using non-uniform refinement to yield to the 3.1 Å half-site map.

To observe DNA dynamics in the Cas1–Cas2/DEDDh half-integration complex, we performed 3DVA^36^ on a subset of particles selected and refined from 2D classification with DNA visible on the leader-distal side of the complex (1,048,353 particles). The filter resolution was 6 Å and the number of modes was 3. To generate Supplementary Video 1, the 3DVA output mode was set to simple and 20 frames, then UCSF ChimeraX was used to generate a vseries. Next, the 3DVA output mode was set to cluster and the number of clusters was set to 20. Each resulting cluster was individually inspected, and two clusters representing maxima of DNA motion along the pitch axis were chosen. The linear structure was derived from 32,722 particles and was processed for non-uniform refinement to give the final 4.1 Å map. The bent structure resulting from initial 3DVA clustering was improved by repetition of the 3DVA workflow with the complete particle set obtained by Topaz picking, then selection and non-uniform refinement of the cluster representing leader-distal DNA in the most bent conformation (53,545 particles total), yielding the final 3.9 Å map.

### Model building and refinement

The initial models of the Cas1 and Cas2/DEDDh were obtained using the AlphaFold 2 program^47^. To build the model of Cas1–Cas2/DEDDh bound to a prespacer with TT PAM complex, the predicted Cas1 and Cas2 monomers were docked independently into the corresponding map with the fitmap tool in UCSF ChimeraX (v.1.2.5)^48^. The DNA models were built de novo. The complex model was refined using rounds of real-space refinement and rigid body fit tools in Coot (v.0.9.4.1)^49^, and real_space_refine tool in Phenix (v.1.19.2-4158)^50^, using secondary structure, Ramachandran, and rotamer restraints. This complex model served as an initial model for other Cas1–Cas2 structures, which were refined in an analogous manner.

### Ligation assays with pCRISPR integration target plasmid

Ligation assays were conducted in integration buffer (20 mM HEPES, pH 7.5, 125 mM KCl, 10 mM MgCl_2_, 1 mM DTT, 0.01% Nonidet P-40 and 10% DMSO). Cas1 (4 μM) and Cas2/DEDDh (2 μM) were pre-complexed for 30 min at 4 °C before addition of DNA substrate (312.5 nM) and integration target pCRISPR (20 ng ml^−1^, ~10 nM) and reacting for 2 h at 37 °C. The reaction was quenched with 0.4% SDS and 25 mM EDTA, treated with proteinase K for 15 min at room temperature, and then treated with 3.4% SDS. The reactions were analysed on a 1.5% agarose gel and visualized using the Typhoon FLA gel imaging scanner.

### Full-site integration assays

Integration assays (50 μl reactions) were conducted in integration buffer (20 mM HEPES, pH 7.5, 125 mM KCl, 10 mM MgCl_2_, 1 mM DTT, 0.01% Nonidet P-40 and 10% DMSO). Cas1 (4 μM) and Cas2/DEDDh (2 μM) were pre-complexed for 30 min at 4 °C before addition of DNA substrate containing BsaI cut sites (312.5 nM) and reacting for 15 min, followed by the addition of the integration target pCRISPR (20 ng ml^−1^, ~10 nM) and incubating for 2 h at 37 °C. The products were purified using the DNA Clean and Concentrator 5 kit (Zymo Research) and eluted with 6 μl water. A gap-filling reaction (20 μl total, 37 °C for 30 min) was conducted with the purified integration products as described previously^37^: 6 μl purified acquisition reaction, 6.5 μl water, 2 μl 10× Taq DNA ligase buffer (NEB), 2 μl dNTP Solution Mix (10 mM stock, NEB), 2 μl Taq DNA ligase (80 U, NEB) and 1 μl T4 DNA polymerase (1 U, NEB). Gap-filling reactions were purified using the Zymo Research kit and eluted with 6 μl water. A Golden-Gate-compatible chloramphenicol selection cassette was generated by PCR with primers encoding BsaI cut sites and purified using the Qiagen MinElute PCR Purification kit. The sequences of primers used are shown in Supplementary Table 2. A Golden Gate cloning reaction was performed using the purified, gap-filled integration products and chloramphenicol selection cassette according to a standard BsaI assembly protocol. The products were purified using the Zymo Research kit and eluted with 6 μl water, and 1 μl was electroporated into DH10B cells (NEB). Electroporated cells were recovered in 975 μl of LB and plated on LB agar containing carbenicillin (100 μg ml^−1^) and chloramphenicol (25 μg ml^−1^). Of the surviving colonies, 95 were sequenced using Sanger sequencing and the sequences were analysed using SnapGene (v.5.0.8).

### CRISPR locus bioinformatic analysis

*Cas2-DEDDh*-containing loci from metagenomic data were identified by determining genomes that contained a CRISPR locus using CRISPRDetect, and coding sequences within 5 kb of the array were extracted^51^. A DEDDh HMM model was built from BLAST searches against the NCBI nr database that were manually verified^52^. The coding sequences were searched against the DEDDh model using hmmsearch with *E* < 1 × 10^−5^ (ref. ^52^). Matches that also contained credible hits to Cas1 and neighbouring other Cas proteins were shortlisted for this work. A preliminary Cas2/DEDDh model was computed using AlphaFold 2 to aid in structure building^47^.

### Statistics and reproducibility

For biochemical experiments, results represent gels of the highest quality. All experiments were generally performed at least in duplicate, although not in the exact same format. Pilot experiments were performed to ensure reproducibility. Measurements were taken from distinct samples. Full-site integration assays were performed by sequencing 95 colonies and counting integration events in biological triplicate. The choice of sample size was made after ensuring reproducibility through pilot experiments. All data points are displayed on the figure panels.

### Reporting summary

Further information on research design is available in the Nature Portfolio Reporting Summary linked to this article.

## Online content

Any methods, additional references, Nature Portfolio reporting summaries, source data, extended data, supplementary information, acknowledgements, peer review information; details of author contributions and competing interests; and statements of data and code availability are available at 10.1038/s41586-023-06178-2.

### Supplementary information

Supplementary Information Supplementary Fig. 1: lightly annotated raw gel files with boxes indicating cropping for main and extended data figures. Supplementary Tables 1 and 2, containing the sequences of proteins used in this study, and oligonucleotides used in this study.

Reporting Summary

Supplementary Video 1 Visualization of repeat DNA flexibility of the Cas1:Cas2-DEDDh half-integration complex using 3-D variability analysis. The PAM side is shown. See Materials and Methods for further detail.

Supplementary Video 2 Visualization of repeat DNA flexibility and DEDDh domain dynamics of the Cas1:Cas2-DEDDh half-integration complex using 3-D variability analysis. The bottom view is shown. See Methods for further details.

### Source data

Source Data Fig. 4

Source Data Extended Data Fig. 2

Source Data Extended Data Fig. 8

## Figures and Tables

**Extended Data Table 1:**
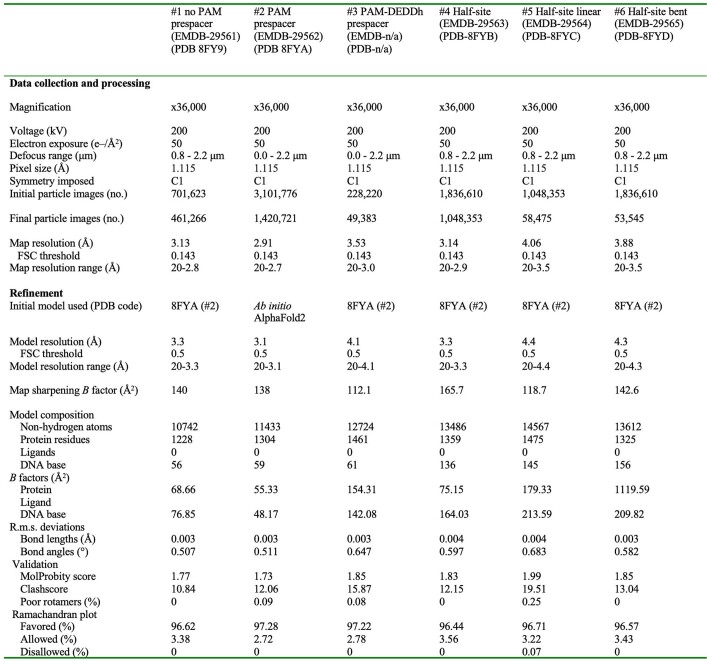
Cryo-EM data collection, refinement and validation statistics

## Data Availability

Atomic models in the Protein Data Bank and the corresponding cryo-EM density maps from the Electron Microscopy Data Bank are available at the following accession codes, respectively: cryo-EM structure of Cas1–Cas2/DEDDh: PAM-deficient prespacer complex (8FY9, EMD-29561); cryo-EM structure of Cas1–Cas2/DEDDh: PAM-containing prespacer complex (8FYA, EMD-29562); cryo-EM structure of Cas1–Cas2/DEDDh: half-site integration complex (8FYB, EMD-29563); cryo-EM structure of Cas1–Cas2/DEDDh: half-site integration complex linear CRISPR repeat conformation (8FYC, EMD-29564); cryo-EM structure of Cas1–Cas2/DEDDh: half-site integration complex with CRISPR repeat bent conformation (8FYD, EMB-29565). The plasmids used in this study are available on reasonable request. Source data are provided with this paper.
