# Supplementary material for: Genome expansion by a CRISPR trimmer-integrase
Source: Nature. 2023 Jun 14;618(7966):855–61. doi: 10.1038/s41586-023-06178-2 (PMC10284694; doi:10.1038/s41586-023-06178-2)
Supplement: Supplementary file 1 — Supplementary Fig. 1: lightly annotated raw gel files with boxes indicating cropping for main and extended data figures. Supplementary Tables 1 and 2, containing the sequences of proteins used in this study, and oligonucleotides used in this study. [file 41586_2023_6178_MOESM1_ESM.pdf]

nature portfolio

https://doi.org/10.1038/s41586-023-06178-2

Supplementary information

Genome expansion by a CRISPR trimmer-integrase

In the format provided by the authors and unedited

Supplementary Figure 1. Uncropped raw gel images

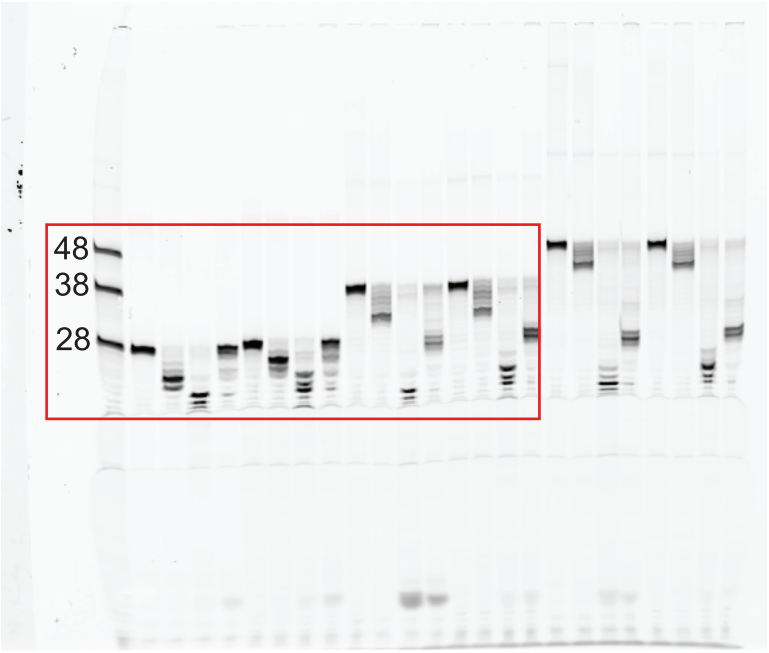

Fig. 1b

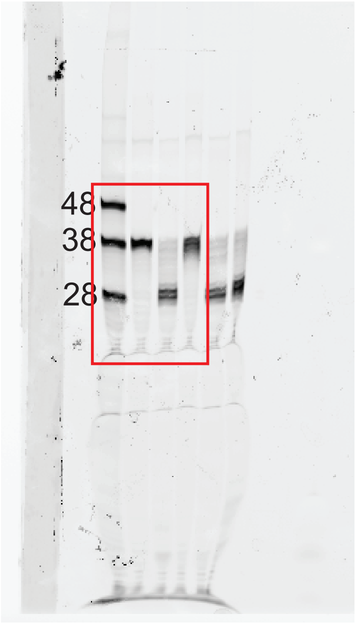

Fig. 1c

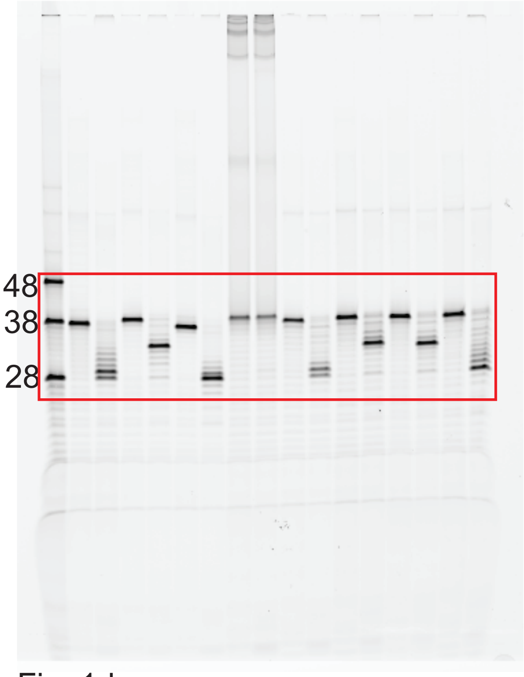

Fig. 1d

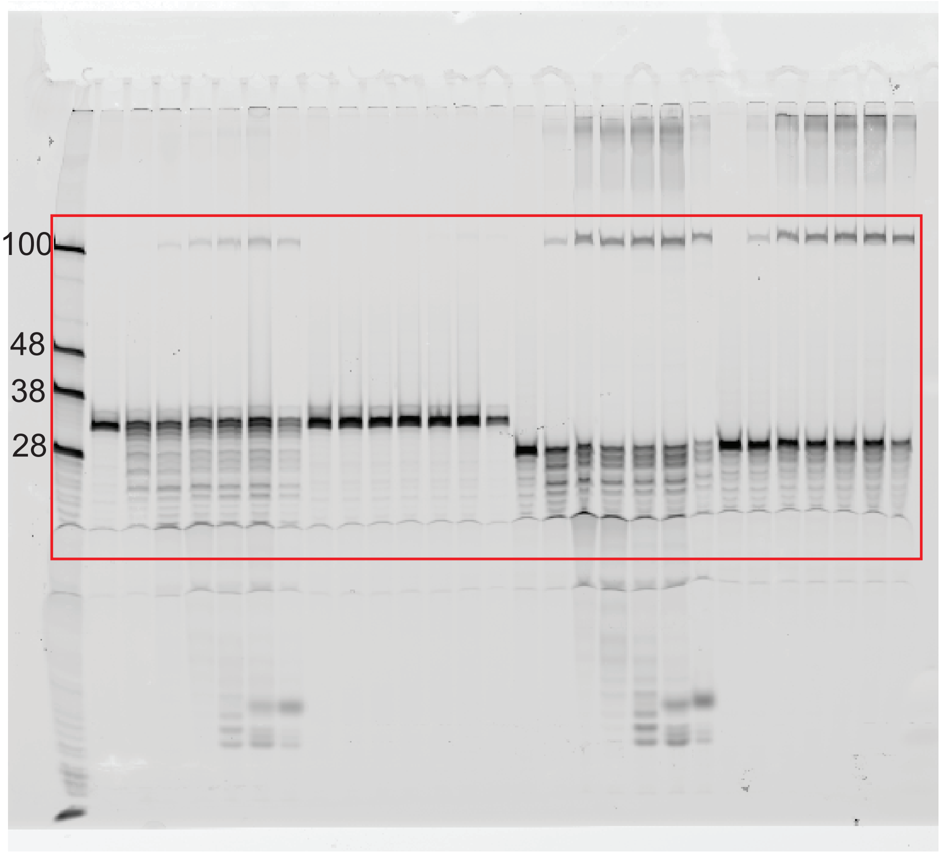

Fig. 3a`

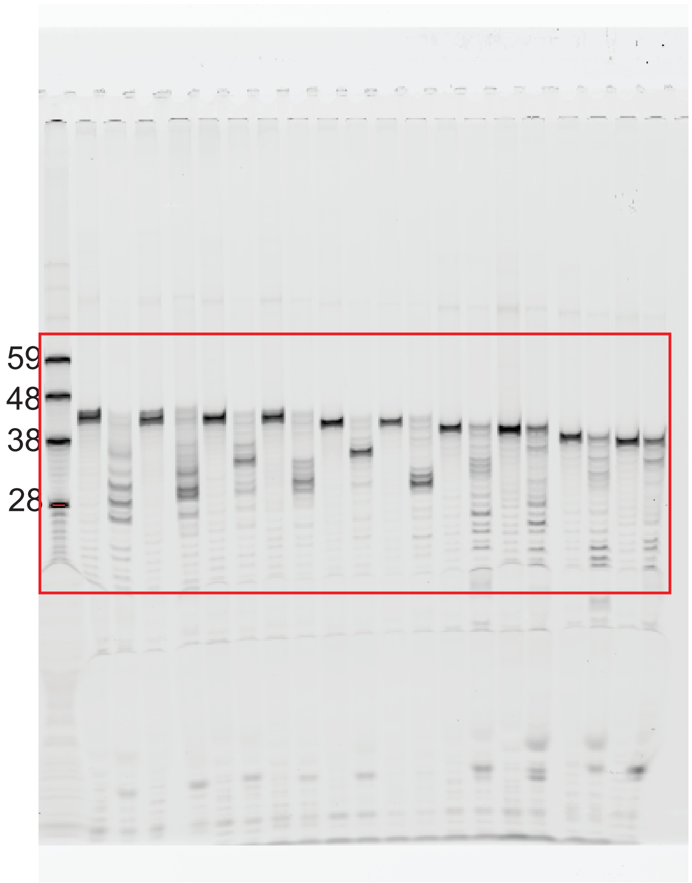

Ext. Data Fig. 1b

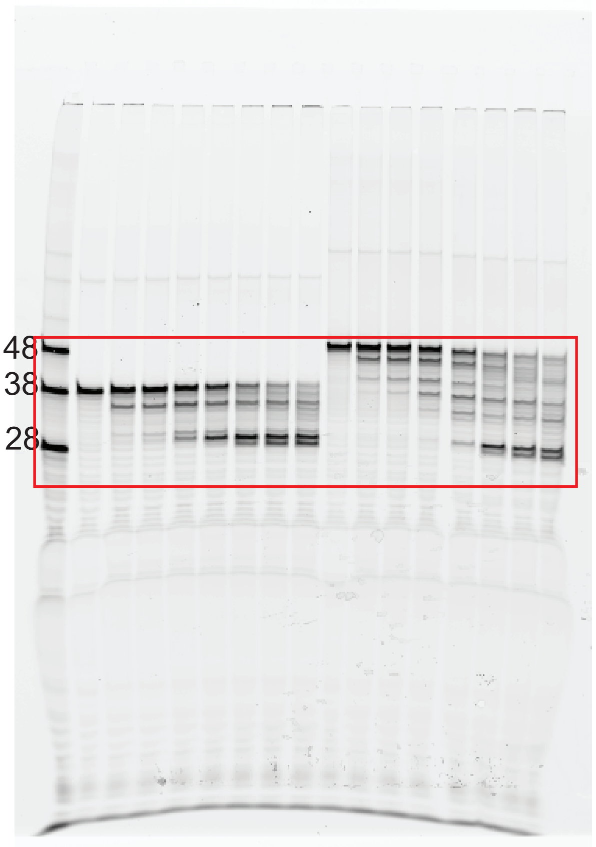

Ext. Data Fig. 2a

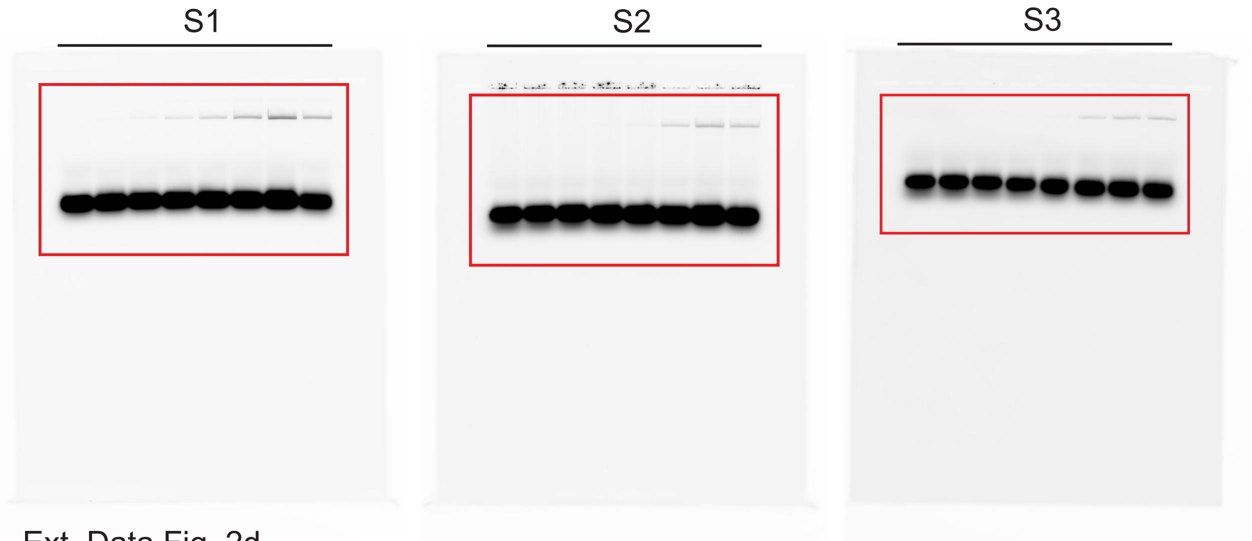

Ext. Data Fig. 2d

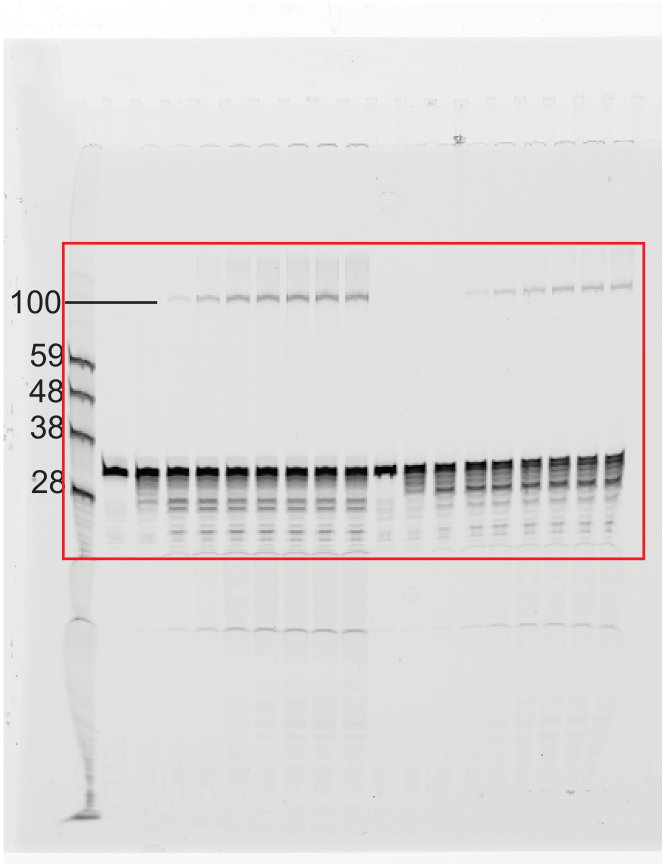

Ext. Data Fig. 6f

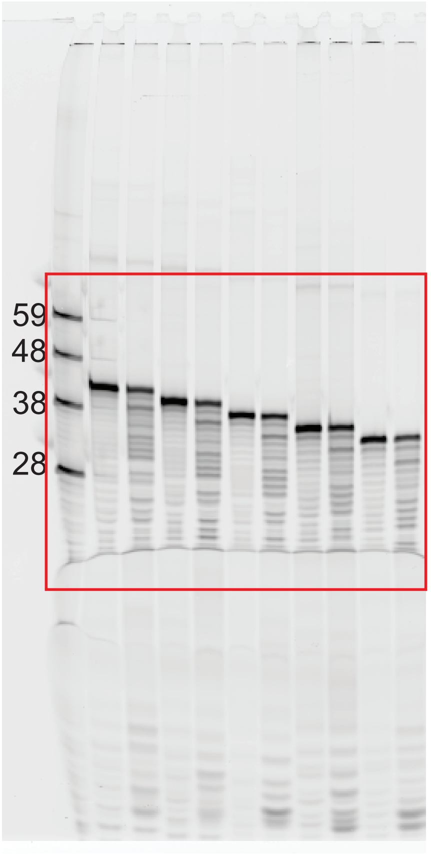

Ext. Data Fig. 8f

Supplementary Table 1. Sequences of the type I-E *Megasphaera NM10_related* Cas1 and Cas2-DEDDh proteins used in this study.

**Protein**	**Sequence**
Cas1	MAGPIIAGKSESSELPRVEDRATFIYIEHAKINRVDSAVTVAEAKG<br>VVRIPAAMIGVLLLGPGTDISHRAVELLGDTGTALVWVGEQGVR<br>YYASGRALARSTRFLVKQAELVTNERSRLRVARRMYQMRFPTE<br>DVSKLTMQQLRSHEGARVRRKYRELSKKYNVPWKKRVYNPDD<br>FAGGDPINQALSAAHVALYGLVHSVVAALGLSPGLGFVHTGHDR<br>SFIYDVADLYKAEITVPIAFAVAAEAEEGQDIGQLARLRTRDAFV<br>DGKILKRMVKDLQTLLEIPEEGQIEAEPLSLWDDKEKLVPYGVN<br>YSEVTSCP*
Cas2-DEDDh	MPMTVITLKNVPQSLRGDLTRWMQEIATGVYVGNFNSRIREYLW<br>RRVQETMGAGEASMCFAARNELGYDFLTENASRSVIDYDGLPLI<br>FIPKEQSAVSDLPKGFSTAAKLHRAHIAGSGKKKEKPIRYVVIDIE<br>TDGKDAKRNHILEIGAIRCEDGKETHFTALISGDAVPPSITKLTGIT<br>ATLLQKEGQEEKKVLTAFREFIGDDDLVGYHVSFDIEFLRQAFKK<br>YGLGYLKNKTHDLLRIVKKEQLFQADYKLETSLQSYGIHKKVPH<br>RALGDAELVKCLAKKLNKF*

Supplementary Table 2. DNA oligonucleotides used in this study. * indicates phosphorothioate bond.

Name/Description	Sequence	Figure
S1_top	actcatgatgcacaagtggttgcgcgtg	1
S1_bottom	gcaaccacttgtgcatcatgagtgatga	1
S2_top<br><br>mixed OH 1 (AA PAM)_top	actcatgatgcacaagtggttgcgcgtgAACCCAGTTG	1, ED1, ED2, ED3
S2_bottom<br><br>Bottom strand of prespacers<br><br>15-nt 3'OH / 23-bp duplex / 15-nt 3'OH (bottom)	gcaaccacttgtgcatcatgagtgatgaAACCCAGTTG	1, 4, 5, ED1, ED2, ED3, ED10
S3_top	actcatgatgcacaagtggttgcgcgtgACCTAGAAGTAACCCAGTTG	ED2
S3_bottom	gcaaccacttgtgcatcatgagtgatgaACCTAGAAGTAACCCAGTTG	ED2
polyA OH (AA PAM)_top	actcatgatgcacaagtggttgcgcgtgAAAAAAAAAA	1, ED3
poly T OH (TT PAM)_top	actcatgatgcacaagtggttgcgcgtgTTTTTTTTTT	1, ED3
poly C OH (CC PAM)_top	actcatgatgcacaagtggttgcgcgtgCCCCCCCCCC	1, ED3
poly G OH (GG PAM)_top	actcatgatgcacaagtggttgcgcgtgGGGGGGGGGG	1, ED3
mixed OH 2 (TT PAM)_top	actcatgatgcacaagtggttgcgcgtgTTTTCAGTTG	1, ED3
mixed OH 3 (TT PAM)_top<br><br>15-nt 3'OH / 23-bp duplex / 15-nt 3'OH (top)	actcatgatgcacaagtggttgcgcgtgTTCCCAGTTG	1, ED3, ED10
mixed OH 4 (TA PAM)_top	actcatgatgcacaagtggttgcgcgtgTACCCAGTTG	1, ED3
Full-site prespacer_top<br><br>Full-site prespacer	aaacggagacctggtctcaatctgcgtgTTCCCAGTTG	4, 5, ED9
Full-site prespacer_bottom<br><br>Full-site prespacer (atg barcode)_bottom	agattgagaccaggtctccgtttcatgaAACCCAGTTG	4, 5, ED9
CryoEM prespacer PAM-deficient strand	actcatgatgcacaagtggttgcgcgtg*AACCCAGTTG	2
CryoEM prespacer PAM-deficient complement strand	gcaaccacttgtgcatcatgagtgatga*AACCCAGTTG	2

CryoEM prespacer PAM-containing strand	actcatgatgcacaagtggttgcgcgtgTTCCC*AGTTG	2
CryoEM prespacer PAM-containing strand complement	gcaaccacttgtgcatcatgagtgatga*AACCCAGTTG	2
Half-site substrate bottom strand (spacer-repeat-leader)	tgcgcgtgggatcacccccgctcgtgcgggaaagacagtaatggattcctttattttcgcccttttacgcttactgac*g*t	3, ED6
Half-site substrate 1 leader strand	acgtcagtaagcgtaaaagggcgaaaataaaggaatccatta*c*t	3, ED6
Half-site substrate protospacer-repeat-spacer strand	agattgagaccaggtctccgtttcatgagtctttcccgcacgagcgggggtgatcccacgcg*c*a	3, ED6
Half-site substrate 1 protospacer + unprocessed PAM strand	aaacggagacctggtctcaatctgcgtgTTCCC	3, ED6
Half-site substrate 2 protospacer strand	aaacggagacctggtctcaatctgcgtg	3, ED6
CryoEM half-site substrate protospacer strand	aaacggagacctggtctcaatctgcgtgT*T*CCC	3
Full-site prespacer_top (with PAM)<br><br>Full-site prespacer (tcg barcode, PAM)_top	aaacggagacctggtctcaatctgtcggTTCCCAGTTG	4, ED9
Full-site prespacer_top (no PAM)<br><br>Full-site prespacer (tcg barcode, no PAM)_top	aaacggagacctggtctcaatctgtcggAACCCAGTTG	4, ED9
Full-site prespacer_bottom<br><br>Full-site prespacer (gat barcode)_bottom	agattgagaccaggtctccgtttcgataAACCCAGTTG	4, ED9
Full-site prespacer (cgt barcode, no PAM)_top	aaacggagacctggtctcaatctgcgtgAACCCAGTTG	4, ED9
D1_top<br><br>SS1	CTGCATCTGGGTATCATCACTCATGATGCACTAGTGGATGCGCGTGATCCTATGCATGA	5
D1_bottom	TCATGCATAGGATCACGCGCATCCACTAGTGCATCATGAGTGATGATACCCAGATGCAG	5
D2_top<br><br>SS2	CTGCATCTGGGTATCATCACTCATGATGCACTAGTGGATGCGCGTGTTCCTATGCATGA	5
D2_bottom	TCATGCATAGGAACACGCGCATCCACTAGTGCATCATGAGTGATGATACCCAGATGCAG	5
SS3	CTGCATCTGGGTATCATCACTCATGATGCACTAGTGGATGTTCGTGATCCTATGCATGA	5
SS4	CTGCATCTGGGTATCATCACTCATGATGCACTA	5

	GTTGATGCGCGTGATCCTATGCATGA	
11-nt 3'OH / 31-bp duplex / 11-nt 3'OH (top)	catcactcatgatgcacaagtggttgcgcgtgTTCCCAGTTG	ED10
11-nt 3'OH / 31-bp duplex / 11-nt 3'OH (bottom)	acgcgcaaccacttgtgcatcatgagtgatgaAACCCAGTTG	ED10
13-nt 3'OH / 27-bp duplex / 13-nt 3'OH (top)	tcactcatgatgcacaagtggttgcgcgtgTTCCCAGTTG	ED10
13-nt 3'OH / 27-bp duplex / 13-nt 3'OH (bottom)	gcgcaaccacttgtgcatcatgagtgatgaAACCCAGTTG	ED10
17-nt 3'OH / 19-bp duplex / 17-nt 3'OH (top)	tcatgatgcacaagtggttgcgcgtgTTCCCAGTTG	ED10
17-nt 3'OH / 19-bp duplex / 17-nt 3'OH (bottom)	aaccacttgtgcatcatgagtgatgaAACCCAGTTG	ED10
19-nt 3'OH / 15-bp duplex / 19-nt 3'OH (top)	atgatgcacaagtggttgcgcgtgTTCCCAGTTG	ED10
19-nt 3'OH / 15-bp duplex / 19-nt 3'OH (bottom)	ccacttgtgcatcatgagtgatgaAACCCAGTTG	ED10
Chloramphenicol selection cassette PCR forward primer	GGCCGGTCTCCAGATtgatcggcacgtaagaggttc	4, ED10
Chloramphenicol selection cassette PCR reverse primer	GGCCTGGTCTCAAAACattctcaccaataaaaaacgcccg	4, ED10
